# Esketamine during anesthesia induction improves early postoperative sleep quality in female patients undergoing laparoscopic surgery: A randomized trial

**DOI:** 10.12669/pjms.42.8.16352

**Published:** 2026-08

**Authors:** Rui Du, Guofang Fei, Junqi Zhang, Qian Cheng

**Affiliations:** 1 Rui Du, Department of Anesthesiology, Huzhou Maternity & Child Health Care Hospital, Huzhou, Zhejiang Province 313000, P.R. China; 2 Guofang Fei, Department of Anesthesiology, Huzhou Maternity & Child Health Care Hospital, Huzhou, Zhejiang Province 313000, P.R. China; 3 Junqi Zhang, Department of Anesthesiology, Huzhou Maternity & Child Health Care Hospital, Huzhou, Zhejiang Province 313000, P.R. China; 4 Qian Cheng, Department of Anesthesiology, Huzhou Maternity & Child Health Care Hospital, Huzhou, Zhejiang Province 313000, P.R. China

**Keywords:** Esketamine, Female, Laparoscopic surgery, Sleep quality

## Abstract

**Objective:**

To evaluate the effect of intravenous esketamine administration during anesthesia induction on postoperative sleep quality in female patients undergoing laparoscopic surgery.

**Methodology:**

This study was designed as a prospective, double-blind, single-center randomized controlled trial and was conducted at Huzhou Maternity & Child Health Care Hospital between December 5, 2025, and January 31, 2026. One hundred ASA I-II female patients (25-50 years) undergoing elective laparoscopic surgery (Dec 2025-Jan 2026) were randomized to control (Group C, n=50) or esketamine (Group S, n=50). Both groups received propofol 2 mg/kg, cisatracurium 0.15 mg/kg, and sufentanil 0.3 μg/kg for induction; Group-S additionally received esketamine 0.3 mg/kg, while Group-C received normal saline. Anesthesia was maintained with propofol (4–6 mg/kg/h) and remifentanil (0.1–0.2 μg/kg/min) via infusion pumps to maintain the Bispectral Index (BIS) between 40 and 60 and end-tidal carbon dioxide (PETCO_2_) between 35 and 45 mmHg. Outcomes included Pittsburgh Sleep Quality Index (PSQI) on postoperative days (POD) one, three, seven; intraoperative sufentanil use; 6/24 h VAS pain scores; 24 hours rescue analgesia rate; adverse events; emergence time; and PACU stay.

**Results:**

Ninety eight patients (Group C: 49; Group S: 49) completed the study. Group-S had lower PSQI scores on POD one and three (P<0.05), reduced intraoperative sufentanil consumption (P<0.05), lower 6/24 hours VAS scores (P<0.05), and lower 24 hours rescue analgesia rate (P<0.05). No significant differences in emergence time, PACU duration, or adverse event rates were observed (P>0.05), with no severe adverse events.

**Conclusion:**

Administration of 0.3 mg/kg esketamine during anesthesia induction reduces opioid consumption, improves early postoperative sleep quality, and decreases sleep disturbance incidence in female laparoscopic patients without increasing adverse event risks.

## INTRODUCTION

Postoperative sleep disturbance (POSD) is a common early post-surgical complication. The severity of POSD is closely related to the duration of surgery, and the extent of tissue trauma and inflammation.^1^ Patients with POSD report difficulty falling asleep, interrupted sleep patterns, and daytime fatigue. Even mild POSD symptoms can lead to prolonged hospital stays and elevated incidence of complications, while more severe POSD has been shown to increase the risks of chronic diseases and cancer.^2^–^4^

Recent research has shown that anesthesia contributes to sleep disturbances by disrupting the rapid eye movement (REM)/nonrapid eye movement (NREM) sleep cycle.^5^ Furthermore, opioids, the most frequently used anesthetic adjuvants, were also associated with increased POSD occurrence.^6^ Opioid use during surgery also increases the risk of respiratory depression, drug tolerance, physiological dependence, and hyperalgesia.^7^ Therefore, there is a need to identify anesthetic adjuvants that can effectively reduce opioid consumption while minimizing the negative impact on sleep quality. Esketamine is a highly selective antagonist of the N-methyl-D-aspartate (NMDA) receptor and is characterized by a rapid onset and a potent, controlled analgesic effect.^8^ Studies have shown that the perioperative use of esketamine can reduce opioid dosage and potentially improve postoperative sleep quality.^9^–^13^ However, while female gender was identified as an independent risk factor for POSD,^14^ evidence regarding the use of esketamine as an anesthetic adjunct in female patients undergoing laparoscopic surgery remains limited.

This study aimed to investigate whether intravenous esketamine during anesthesia induction improves postoperative sleep quality in female patients undergoing laparoscopic surgery. The results may offer an alternative approach to traditional opioid-based pain management strategies.

## METHODOLOGY

This was a prospective, double-blind, single-center randomized controlled trial conducted at Huzhou Maternity & Child Health Care Hospital. Female patients (aged 25–50 years, ASA physical status I–II) scheduled for elective laparoscopic surgery at our institution between December 5, 2025, and January 31, 2026, were included. Written informed consent was obtained from all participants before randomization.

Sample size estimation was derived from preliminary pilot data obtained from 20 patients, wherein we noted difference in POD1 PSQI scores (Group S: 5.9±1.4 vs Group C: 6.8±1.5). Since pilot studies with small sample sizes can overestimate effect magnitude, the calculated standardized effect size was considered exploratory and was used only to guide trial planning. Using a two-sided α of 0.05 and a statistical power of 80% (β=0.20), the minimum required sample size was required to 41 patients per group. Therefore, to account for potential participant dropout and protocol deviations, we included 50 patients in each group.

### Ethical approval and Registration:

This prospective, double-blind, randomized controlled trial (registered at the Chinese Clinical Trial Registry under ID: ChiCTR2500113859) received approval from the Ethics Committee of Huzhou Maternity and Child Health Care Hospital (Approval No.: 2025-J-092; dated November 30, 2025).

One hundred female patients (ASA I–II, aged 25–50 years) scheduled for elective laparoscopic surgery were randomized into Group-C (n=50) or Group-S (n=50). Randomization was performed using a computer-generated random sequence (block size six), with assignments concealed in sequentially numbered, opaque, sealed envelopes. Specifically, an independent biostatistician who did not participate in patient recruitment, clinical management, or data analysis generated the randomization sequence. Eligible patients were screened and enrolled by two attending anesthesiologists. All outcome assessors, data collectors, and statistical analysts remained strictly blinded to group allocation throughout the entire trial to reduce detection bias.

### Inclusion criteria:

- Age 25-50 years, ASA I-II.
- Preoperative PSQI score <5 (exclude baseline sleep disorders).

### Exclusion criteria:

- History of psychiatric disorders/chronic pain.
- Long-term sedative/opioid use.
- Known allergy to study drugs.
- Body mass index (BMI)≥30 kg/m^2^.
- Anxiety and Depression Scale (HADS) score ≥8.

### Study protocol:

Following a standardized minimum 8-hour preoperative fasting protocol (including both solids and clear liquids), patients were transferred to the operating theater. Continuous physiological monitoring was established using multiparameter devices: Non-invasive blood pressure (NIBP) measurements were automated at five minute intervals; 5-lead electrocardiogram (ECG) monitored cardiac rhythm and ST-segment changes; pulse oximetry (SpO_2_) tracked peripheral oxygenation saturation; bispectral index (BIS) sensors (Aspect Medical Systems) were applied to the forehead to quantify depth of anesthesia. Baseline hemodynamic parameters (systolic/diastolic BP, heart rate, SpO_2_) were recorded after five minutes of supine rest.

Anesthesia induction was performed as follows: intravenous propofol 2 mg/kg over 30 seconds, cisatracurium 0.15 mg/kg, and sufentanil 0.3 μg/kg administered sequentially. Group-S received an additional dose of esketamine, 0.3 mg/kg, IV over 60 seconds. Group-C received an equivalent volume of 0.9% saline (placebo). Drug administration was performed by anesthesiologists blinded to group assignment using identical 10 mL opaque syringes prepared by an independent research nurse who did not participate in patient anesthesia, data collection or statistical analysis.

For anesthesia maintenance, continuous infusion of propofol (4-6 mg/kg^/^h) and remifentanil (0.1-0.2 μg/kg^/^min) was titrated to maintain mean arterial pressure (MAP) within ± 20% of baseline values, BIS 40–60, and end-tidal carbon dioxide (PETCO_2_) 35–45 mmHg. All patients were housed in individual wards, with ambient noise levels maintained at ≤ 45 dB (verified by daily sound meter measurements). Fixed sleep-wake cycles were implemented, with lights off from 22:00 to 06:00 for three consecutive preoperative days, during which nursing staff ensured compliance through hourly room checks.

### Environmental Standardization:

### Postoperative phase:

During the first 72 hours after surgery, patients were housed in individual sound-attenuated wards. Environmental controls included:

### Noise suppression:

Continuous ambient noise ≤ 45 dB verified by calibrated sound level meter measurements at 08:00 /16:00/ 22:00 daily.

### Light-dark cycles:

Strict photoperiod enforcement with lights-off from 22:00 to 06:00, using blackout curtains and timed electrical controls.

### Compliance monitoring:

Nursing staff conducted hourly room checks (22:00-06:00) and documented light exposure and noise violations.

### Outcome measures:

The primary outcome was subjective sleep quality. The PSQI was originally designed to assess sleep status over a one-month period for chronic sleep assessment; nevertheless, multiple recent perioperative clinical trials have validated its modified application for short-term postoperative sleep evaluation (POD 1, 3, and 7) by focusing on subjective sleep experience of the previous single night before assessment.^15^,^16^

In this study, a trained, blinded assessor conducted structured interviews and modified the PSQI time scale to evaluate patients’ sleep quality within 24 hours before each assessment time point, which is a widely accepted method for acute postoperative sleep disturbance evaluation in surgical populations. Additionally, preoperative baseline PSQI scores were recorded to verify the homogeneity of sleep status between the two groups before intervention. PSQI was evaluated via structured interview by a trained, blinded assessor who had no access to patient grouping information and intraoperative medical records. All pain scale scoring, adverse event recording and postoperative follow-up were completed by independent blinded researchers to avoid assessment bias.

### Secondary outcomes included:

### Pain intensity:

VAS scores (0-10 cm) assessed at rest and during movement (e.g., coughing) at 6h and 24 hours postoperatively.

### Analgesic Consumption:

[Intraoperative sufentanil (μg)], Postoperative 24 hours and 48 hours opioid consumption (converted to morphine milligram equivalents - MME).^17^

24 hours and 48 hours rescue analgesia rate (%); Recovery parameters: Emergence time (min, defined as time from cessation of anesthetics to extubation), PACU stay duration (min).

### Safety outcomes:

nausea/vomiting (N/V): frequency, severity (Grade 1-3 Grade-1: Nausea only without vomiting; Grade-2: Retching or vomiting; Grade-3: Intractable nausea or vomiting),^18^ duration, and antiemetic use (Ondansetron mg), neuropsychiatric events, dizziness (incidence, severity), hallucinations/dissociation (assessed using the Brief Psychiatric Rating Scale (BPRS) at 1h and 6h post-PACU admission, incidence and intensity recorded), and overall adverse event incidence (%) documented up to POD seven.

### Statistical analysis:

Statistical analysis was performed using SPSS 26.0. The Shapiro-Wilk test was applied to assess the normal distribution of continuous variables. Normally distributed data were presented as mean ± standard deviation (mean ± SD) and compared using independent-samples t-tests, while non-normally distributed data were analyzed with the Mann-Whitney U test. Categorical data were expressed as frequency (%) and analyzed with χ² tests or Fisher’s exact tests. Considering multiple endpoints including repeated PSQI measurements at three time points, serial VAS pain scores, and various secondary analgesic and safety outcomes, the Bonferroni correction was performed to control the Type-I error caused by multiple statistical comparisons. A two-sided P < 0.05 was considered statistically significant.

## RESULTS

Ninety-eight patients were included in the final analysis (Group C: n = 49; Group-S: n = 49). Demographic characteristics and procedural times were comparable between the two groups, with no statistically significant difference (P>0.05; Table-I). Compared to Group- C, Group-S demonstrated significantly lower PSQI scores on POD 1 (P < 0.05) and POD 3 (P < 0.05). No significant differences in PSQI scores between the groups was observed on POD 7 (P > 0.05; Table-II).

**Table-I:** Patient Characteristics and Procedural Data (mean±SD, n=49).

Group	Age years	BMI kg/m^2^	ASA I/II	Anesthesia Time min	Surgery Time min
S	37.5±5.0	24.2±2.5	25/ 24	45.0±10.0	42.0±8.0
C	38.0±4.8	23.8±2.6	26/ 23	46.0±9.5	43.0±7.5
Statistical Values	0.52	0.78	0.08	0.52	0.60
P value	0.605	0.436	0.777	0.603	0.551

**Table-II:** PSQI Scores (mean±SD, n=49).

Group	PSQI scores			
	Pre-op Day 1	POD 1	POD 3	POD 7
S	3.5±1.3	4.2±1.3	3.5±1.1	3.2±0.9
C	3.7±1.4	6.8±1.5	5.9±1.4	3.5±1.0
Statistical Values	0.7328	9.52	9.18	1.58
P value	>0.05	<0.001	<0.001	0.118

Patients in Group S exhibited significantly reduced intraoperative sufentanil consumption (P < 0.05), lower VAS pain scores at both six hours (P < 0.05) and 24 hours (P < 0.05) postoperatively, and a decreased 24 hours rescue analgesia rate (P < 0.05) compared to Group-C (Table-III). There were no significant differences between groups in emergence time, PACU length of stay, or the overall incidence of adverse events (P > 0.05; Table-IV). No severe adverse events occurred in either group.

**Table-III:** Analgesia Outcomes (mean±SD, n=49).

Group	Sufentanil μg	VAS 6 hours	VAS 24 hours	Rescue Analgesia Rate (%)
S	15.6±3.2	2.1±0.7	1.8±0.6	8.2%
C	22.4±4.1	3.2±0.9	2.7±0.8	24.5%
Statistical Values	9.42	6.75	6.28	5.02
P value	<0.001	<0.001	<0.001	0.025

**Table-IV:** Safety Outcomes (mean±SD, n=49).

Group	Emergence min	PACU Stay min	PONV	Dizziness	Hallucination	Adverse Events %
S	8.5±1.7	46.8±5.9	6.1%	4.1%	0	10.2%
C	8.2±1.5	45.3±6.2	8.2%	6.1%	0	14.3%
Statistical Values	0.93	1.23	0.00	0.00		0.39
P value	0.355	0.222	>0.05	>0.05		0.532

## DISCUSSION

This study demonstrated that in female patients undergoing laparoscopic surgery, administration of 0.3 mg/kg esketamine during anesthesia induction reduces opioid consumption, improves early postoperative sleep quality, without increasing the risk of adverse events.

Compared with open surgery, laparoscopic surgery theoretically improves sleep due to reduced tissue trauma and shorter recovery periods.^19^ However, postoperative pain and discomfort caused by pneumoperitoneum-induced diaphragmatic irritation and residual carbon dioxide insufflation, along with accumulated sleep deprivation from rapid surgical turnover schedules and pre-operative fasting protocols, may substantially offset the minimally invasive advantages of laparoscopic surgery.^20^

Female patients may have several risk factors that may contribute to deterioration in sleep quality. Postoperative stress was shown to induce hormonally mediated imbalances, such as elevated cortisol that suppresses melatonin synthesis, estrogen fluctuations that disrupt circadian rhythms, leading to increased difficulty falling and staying asleep, and sleep fragmentation.^21^ Furthermore, female patients have inherently lower pain perception thresholds due to neuroendocrine differences in pain pathway modulation, leading to higher postoperative opioid use to achieve adequate analgesia.^22^ Increased opioid consumption, in turn, may amplify drug-related REM sleep suppression and alter sleep quality.^23^ Additional important factors to consider, include disproportionate family caregiving responsibilities of female patients, their household dynamics, unresolved preoperative emotional distress, etc. These psychosocial factors may further impact the duration and sleep quality, extending them beyond the acute recovery phase.^24^

This study showed that esketamine use was associated with significantly improved early postoperative sleep quality, particularly on POD one and three. Patients in Group-S exhibited markedly reduced intraoperative sufentanil use compared to Group-C, receiving standard opioid-based analgesia. Reduced opioid exposure may have contributed to the improved postoperative sleep quality observed in Group-S. Notably, the between-group differences in PSQI and VAS scores were not only statistically significant but also clinically meaningful; the magnitude of these score reductions was sufficient to alleviate subjective sleep discomfort and postoperative pain perception in daily clinical practice. Furthermore, patients in Group-S consistently showed lower dynamic and resting pain scores at six hours and 24 hours after surgery, as well as significantly fewer requests for rescue analgesia. These benefits were achieved without introducing additional risks of hemodynamic instability, psychotomimetic reactions, or delayed milestones of recovery.

Postoperative sleep disturbances are largely attributable to opioid analgesic–related alterations in fundamental sleep architecture. On the one hand, activation of μ-opioid receptors in the central nervous system potently inhibits REM sleep, which is associated with cognitive restoration and emotional processing.^25^ On the other hand, opioids induce significant fragmentation of δ-wave (slow-wave) sleep, the deep sleep stage essential for physical restoration and recovery, markedly impairing overall sleep continuity and its restorative quality.^26^,^27^ As a potent S-enantiomer NMDA receptor antagonist, esketamine has a distinct pharmacokinetic profile characterized by an extremely rapid onset of action. It achieves peak plasma concentration within 1–2 minutes after intravenous administration and has a relatively short duration of effect, as evidenced by a plasma elimination half-life of approximately two hours.^28^

Beyond its direct analgesic and opioid-sparing actions, perioperative esketamine application has demonstrated efficacy in alleviating symptoms of anxiety and depression in the surgical context.^29^ Recent studies suggest that esketamine may modulate central nervous system function, having a mood-stabilizing effect. Previous studies have shown that esketamine may also have beneficial effects on postoperative mood and emotional recovery, which could indirectly influence sleep quality.^30^,^31^ All patients in this study reported similar sleep quality metrics by POD 7. It is plausible that esketamine’s inherently short half-life leads to a rapid decline in its effects on sleep pathways. Furthermore, baseline sleep patterns usually begin to re-establish in many patients approximately one week after surgery, irrespective of the intervention.

When administered at the low-dose regimen of 0.3 mg/kg used in this protocol, esketamine showed acceptably low rates of typical transient adverse events, such as nausea/vomiting and dizziness. This study found that the esketamine and the control group of patients had comparable emergence time from anesthesia, duration of stay in the PACU, and the overall incidence of reported adverse events. Notably, no instances of severe adverse psychological events, such as hallucinations or emergence delirium, were reported in either study group during the observation period.

### Strengths of the study:

First, it focuses on female laparoscopic patients, as female gender is an independent risk factor for postoperative sleep disturbance. By targeting this population, the findings provide specific insights into the efficacy of esketamine in high-risk groups. Second, this study minimizes bias through strict inclusion criteria (ASA I-II, 25-50 years) and random allocation. Third, the outcome measures are comprehensive, including the PSQI at multiple time points (POD 1, 3, 7), intraoperative opioid consumption, pain scores, and adverse events, ensuring a comprehensive assessment of the impact of esketamine on sleep quality and perioperative safety. Finally, the use of a low dose (0.3 mg/kg) of esketamine demonstrates clinical feasibility, as it reduces opioid requirements, improves sleep without increasing adverse events, supporting its practical application in routine clinical settings.

### Limitations:

Despite these strengths, several areas warrant further investigation. First, this is a single-center clinical trial conducted in a single maternity and child health care hospital, and the study only enrolled female patients, which greatly limits the generalizability and extrapolation of the research findings. Second, the long-term effects of esketamine on patients’ sleep quality remain unclear; future studies should extend the follow-up period. Third, the mechanism by which esketamine modulates sleep and its interaction with NMDA receptors and neurotransmitter systems require further exploration. Fourth, subgroup analyses based on different surgical types or patient characteristics could clarify the efficacy of esketamine in specific subgroups. Fifth, the original PSQI scale has been validated for one month long-term sleep assessment, but its application at acute postoperative time points (POD 1 and POD 3) has methodological limitations. Although we referred to published perioperative trials using the same modified protocol, there is still a lack of a unified gold standard for short-term PSQI application. Future research can combine objective monitoring indicators such as activity maps or polysomnography to validate postoperative acute sleep changes. Moreover our sample size calculation was based on a relatively small pilot cohort, which may have led to overestimation of the treatment effect size. Therefore, larger-scale multicenter trials are needed to validate these findings and establish optimal dosing regimens for different clinical scenarios.

## CONCLUSION

Intravenous administration of 0.3 mg/kg esketamine during anesthesia induction is associated with a considerable improvement in early postoperative sleep quality in female patients undergoing laparoscopic surgery. It may improve postoperative sleep quality without increasing the risk of adverse events. Present evidence is based on only female participants, using subjective sleep assessment and is devoid of long-term follow-up. Further robust studies overcoming these limitations are needed for better evidence.

## References

[1] Rampes S, Ma K, Divecha YA, Alam A, Ma D (2019). Postoperative sleep disorders and their potential impacts on surgical outcomes. J Biomed Res.

[2] Zhang W, Gao T, Liu F, Zhang H, Wang S (2023). Perioperative sleep disorders in gynaecological daycase surgery patients and analysis of risk factors: protocol for a cross-sectional study. BMJ Open.

[3] Xu W, Zheng Y, Suo Z, Yang Y, Yang J, Wang Q (2024). Potential vicious cycle between postoperative pain and sleep disorders: A bibliometric analysis. Heliyon.

[4] Kulpatcharapong S, Chewcharat P, Ruxrungtham K, Gonlachanvit S, Patcharatrakul T, Chaitusaney B (2020). Sleep Quality of Hospitalized Patients, Contributing Factors, and Prevalence of Associated Disorders. Sleep Disord.

[5] Eckert DJ, Yaggi HK (2022). Opioid Use Disorder, Sleep Deficiency, and Ventilatory Control: Bidirectional Mechanisms and Therapeutic Targets. Am J Respir Crit Care Med.

[6] Pinheiro MF, Relva IC, Costa M, Mota CP (2024). The Role of Social Support and Sleep Quality in the Psychological Well-Being of Nurses and Doctors. Int J Environ Res Public Health.

[7] Hah JM, Bateman BT, Ratliff J, Curtin C, Sun E (2017). Chronic Opioid Use After Surgery: Implications for Perioperative Management in the Face of the Opioid Epidemic. Anesth Analg.

[8] Wang GR, Wu Q, Liu WP, Jing YM (2021). Effect of Oxycodone hydrochloride combined with Dexmedetomidine on quality of recovery and stress response after general anesthesia in patients who had Laparoscopic Cholecystectomy. Pak J Med Sci.

[9] Sun L, Zhao Y, Li Y, Zhai W, Gao F, Yin Q (2023). Effect of continuous subanesthetic esketamine infusion on postoperative fatigue in patients undergoing laparoscopic radical resection for colorectal cancer: a randomized controlled study. Am J Cancer Res.

[10] Wang H, Wang L, Gao J, Zhou F (2025). Effect of intravenous esketamine on postoperative sleep disturbance, anxiety, and depression in elderly patients undergoing laparoscopic abdominal surgery: a randomized controlled trial. BMC Geriatr.

[11] Zhan Y, Zhang Y, Liu Z, Yin Z, Wang D, Xie X (2025). Effects of Subanesthetic Esketamine on Postoperative Sleep Quality Through EEG Analysis in Breast Cancer Patients: A Randomized Clinical Trial. Drug Des Devel Ther.

[12] Wang Y, Xu J, Zhou X, Che L, Qiu M, Liu Y (2026). Esketamine/Ketamine: Dual-Action Mechanisms and Clinical Prospects beyond Anesthesia in Psychiatry, Immunology, and Oncology. Adv Sci (Weinh).

[13] Wu Y, Yang Y, Wang X, Sun L, Hei Y, Zou Y (2025). Effect of intraoperative low-dose esketamine infusion on postoperative sleep disturbance after laparoscopic cholecystectomy: a randomized clinical trial. BMC Anesthesiol.

[14] Wang H, Luo M, Yang Y, Li S, Liang S, Xu R (2024). Gender differences in postoperative pain, sleep quality, and recovery outcomes in patients undergoing visual thoracoscopic surgery. Heliyon.

[15] Wang SW, Zhang XY, Yu YY, Ye XM, Wang Y, Wang L (2026). Association of dexmedetomidine use during combined spinal-epidural anesthesia with postoperative sleep quality and anxiety in cesarean section: a prospective cohort study. BMC Anesthesiol.

[16] Wang Q, Zhao Y, Ling B, Chen X, Xie Y, Zhao H (2025). Efficacy and safety of hydromorphone for postoperative patient-controlled intravenous analgesia for patients undergoing orthopedic surgery: a randomized, double-blinded controlled trial. Front Med (Lausanne).

[17] Adams MCB, Sward KA, Perkins ML, Hurley RW (2025). Standardizing research methods for opioid dose comparison: the NIH HEAL morphine milligram equivalent calculator. Pain.

[18] National Cancer Institute (2025). Common Terminology Criteria for Adverse Events (CTCAE) Version 6.0 [Internet].

[19] Sipilä RM, Kalso EA (2021). Sleep Well and Recover Faster with Less Pain-A Narrative Review on Sleep in the Perioperative Period. J Clin Med.

[20] Duran MK, Öztürk Ş (2024). The effect of shoulder massage on shoulder pain and sleep quality in patients after laparoscopic cholecystectomy: a randomized controlled trial. BMC Nurs.

[21] Feng YZ, Chen JT, Hu ZY, Liu GX, Zhou YS, Zhang P (2023). Effects of Sleep Reactivity on Sleep Macro-Structure, Orderliness, and Cortisol After Stress: A Preliminary Study in Healthy Young Adults. Nat Sci Sleep.

[22] Da-Cas CD, Valesan LF, Nascimento LP do, Denardin ACS, Januzzi E, Fernandes G (2024). Risk factors for temporomandibular disorders: a systematic review of cohort studies. Oral Surg Oral Med Oral Pathol Oral Radiol.

[23] Moore JT, Kelz MB (2009). Opiates, sleep, and pain: the adenosinergic link. Anesthesiology.

[24] Azizoddin DR, Soens MA, Beck MR, Flowers KM, Edwards RR, Schreiber KL (2023). Perioperative Sleep Disturbance Following Mastectomy: A Longitudinal Investigation of the Relationship to Pain, Opioid Use, Treatment, and Psychosocial Symptoms. Clin J Pain.

[25] Dhaliwal A, Gupta M (2025). Physiology, Opioid Receptor. In: StatPearls.

[26] Butris N, Tang E, Pivetta B, He D, Saripella A, Yan E (2023). The prevalence and risk factors of sleep disturbances in surgical patients: A systematic review and meta-analysis. Sleep Med Rev.

[27] Scharf MT, Kelz MB (2013). Sleep and Anesthesia Interactions: A Pharmacological Appraisal. Curr Anesthesiol Rep.

[28] Perez-Ruixo C, Rossenu S, Zannikos P, Nandy P, Singh J, Drevets WC (2021). Population Pharmacokinetics of Esketamine Nasal Spray and its Metabolite Noresketamine in Healthy Subjects and Patients with Treatment-Resistant Depression. Clin Pharmacokinet.

[29] Wang J, Feng Y, Qi Z, Li J, Chen Z, Zhang J (2024). The role and mechanism of esketamine in preventing and treating remifentanil-induced hyperalgesia based on the NMDA receptor-CaMKII pathway. Open Life Sci.

[30] Chen Y, He J, Huang R, Pan Z, Zhong M, Zhang W (2025). Effects of the subanesthetic dose of esketamine on postoperative sleep quality in patients undergoing modified radical mastectomy: a randomized, double-blind controlled trial. Front Med (Lausanne).

[31] Qiu D, Wang XM, Yang JJ, Chen S, Yue CB, Hashimoto K (2022). Effect of Intraoperative Esketamine Infusion on Postoperative Sleep Disturbance After Gynecological Laparoscopy. JAMA Netw Open.

